# Influence of energy drink consumption on perceived quality of life in young adults and adult population

**DOI:** 10.3389/fnut.2025.1662098

**Published:** 2025-12-03

**Authors:** Miriam Otero-Requeijo, Sergio Veiga-Rodeiro, Lorena Belda-Ferri, Víctor José Villanueva-Blasco

**Affiliations:** 1 Faculty of Health Sciences, Valencian International University, Valencia, Spain; 2 Research Group in Health and Psycho-Social Adjustment (GI-SAPS), Valencian International University, Valencia, Spain

**Keywords:** energy drinks, quality of life, WHOQOL-BREF, public health, young adult, adults, prevention

## Abstract

**Background:**

Energy drink (ED) use is common among young adults and adult populations and may be linked to poorer quality of life (QoL). This study examined the prevalence of ED consumption and its association with QoL in Spanish adults.

**Method:**

A cross-sectional design was used with a sample of 1,146 participants aged 18 to 54 years, recruited by convenience sampling through university mailing lists and social media. The web-based questionnaire collected self-reported their energy drink and other substance use and their quality of life (WHOQOL-BREF). Multivariable linear and ordinal logistic regressions were stratified by sex (given a significant sex×ED interaction) and adjusted for age, living arrangement, education, occupation, self-rated health, and past-month use of alcohol, tobacco, and cannabis.

**Results:**

Among 1,146 participants, ED use was 62.7% ever, 21.3% past 30 days, and 5.7% daily. Although most associations were not statistically significant, point estimates suggested lower QoL among ED consumers. In men, daily ED use was associated with lower psychological (mental health) domain scores (*β* = −8.72; 95% CI − 17.00 to −0.45; *p* = 0.039). In women, daily ED consumers had higher odds of perceiving a less healthy environment (OR = 2.58; 95% CI 1.28–5.17; *p* = 0.008). Additional item-level analyses indicated higher odds of negative mood (men: OR = 3.53; 95% CI 1.28–9.75; *p* = 0.015) and dissatisfaction with self (men: OR = 2.71; 95% CI 1.07–6.89; *p* = 0.036) among daily users.

**Conclusion:**

ED consumption is associated with poorer QoL indicators, particularly mental health in men and environmental perceptions in women. Findings support targeted prevention and proportionate regulation (e.g., labeling, marketing oversight), with attention to sex and age profiles within adult populations.

## Introduction

Energy drinks (EDs) are non-alcoholic beverages with high caffeine and sugar, often combined with stimulants such as taurine, ginseng, and guarana (1, 2). The category has expanded rapidly worldwide, with ~200 brands in more than 140 countries (3). Caffeine content varies widely (≈50–505 mg/serving), compared with ~90 mg in 250 mL of coffee, ~50 mg in 250 mL of tea, and ~34 mg in 500 mL of cola (4, 5). EFSA proposes daily “generally safe” intakes of ~3 mg/kg for children/adolescents and ~5.7 mg/kg for healthy adults, levels that ED consumption can meet or surpass, sometimes doubling or tripling these thresholds (4).

Across studies, ED use is more prevalent in males than females at most ages (6–12). Age gradients are less consistent. Some studies have found that consumption levels increase with age (9, 13–19), while others report the opposite (20–22).

ED consumption typically begins early, especially during adolescence, driven by intensive advertising, perceived “functional” benefits, and the social normalization of the product (12, 23). Promotional campaigns emphasize that EDs improve performance and alertness, increase stamina and energy, reduce fatigue, and optimize overall functioning, while also appealing to sensation-seeking and masculine traits (24–26). Consistent with this positioning, the most frequently reported motives among adolescents and university students include studying and enhancing academic performance, engaging in sports and improving physical performance, “boosting energy/alertness,” staying awake, and, to a lesser extent, taste (9, 10, 12, 24, 25, 27, 28).

Gender-targeted marketing, specifically aimed at young males, has been identified as a key determinant of the higher ED consumption observed among adolescents and young adults (12, 24, 25, 29, 30). Consistent with this, the highest prevalences are found in these groups, with adolescents as the main consumers, followed by young adults (6, 7).

Scientific evidence links ED consumption with poorer health and well-being indicators. Population and student studies show that high or frequent consumption is associated with more health complaints and worse self-rated health (9, 15, 16, 31–33). In particular, Khouja et al. (9) observed that those who consume EDs ≥ 5 days/week have a higher risk of poor psychological and physical well-being and are four times more likely to report low academic and overall well-being. Related findings link ED use with poorer perceived health (15, 16, 33, 34). Frequent consumption is associated with mental health problems (anxiety, depressive symptoms, stress, suicidal ideation and behaviors) and with increases in ADHD symptoms and behavioral problems (35–37). In the physical domain, reported effects include sleep disturbances (shorter duration, poorer quality) and greater psychological distress (9, 11), as well as physiological effects, especially among adolescents, such as palpitations, elevated blood pressure, increased arterial stiffness, and signs of insulin resistance (38–40).

In Spain, ED consumption is common among both young adults and adults. According to the Spanish Survey on Alcohol and Other Drugs, EDADES (49), 14.2% of the population aged 15–64 consumed EDs in the past 30 days. The highest prevalence is observed in 15–24-year-olds (37.6%; 42.8% in men and 32.1% in women), a band that overlaps the lower end of our adult sample (18–24), indicating elevated use among younger adults. Moreover, mixing EDs with alcohol in leisure settings is common, a practice that increases associated risks (49).

Socioculturally, consumption is linked to the pursuit of academic and athletic performance, prolonged wakefulness, and nightlife, patterns widely described among university students and young adult healthcare trainees in Spain (41, 42, 49).

The regulatory framework requires specific high-caffeine warnings on labels (Regulation [EU] No 1169/2011) but does not set a nationwide minimum purchase age. Some autonomous communities have proposed or adopted restrictions (e.g., initiatives in Galicia and limitation measures in Asturias), reflecting social concern (49). Internationally, several countries apply stricter measures: a sales ban to minors in Sweden, pharmacy-only dispensing in Norway, and legal limits on caffeine/taurine content in Germany and Denmark (3). In the United Kingdom, major retail chains have voluntarily restricted sales to those under 16 (69).

Furthermore, the systematic review by Ajibo et al. (6) consistently reports an association between ED consumption and the use of substances such as alcohol, tobacco, and cannabis, highlighting that individuals who consume EDs are more likely to use these other substances, especially when ED consumption is frequent.

The evidence suggests that regular ED consumption is associated with poorer quality of life through three main pathways: sleep disturbances, deteriorations in mental health, and behavioral changes. A cross-sectional study in Norway involving more than 53,000 university students showed a dose–response relationship between consumption frequency and shorter/less efficient sleep, as well as higher insomnia rates (43). Given that adequate sleep is a key determinant of quality of life, this association is clinically relevant (43).

At the longitudinal level, a follow-up study of young Australian adults found that increases in ED consumption predicted greater symptoms of depression, anxiety, and stress, especially among men, suggesting a sustained impact on psychological well-being (44). Reviews corroborate these trends. Although there may be immediate effects on alertness, frequent intake is linked to higher stress, anxiety, and depressive symptoms (17, 18) and to an overall decline in well-being and quality of life (45). In Europe, behavioral effects (mixing with alcohol, impulsivity) and physiological effects (tachycardia, hypertension) have also been described, with potential social and functional repercussions (46).

Given the evidence presented, it is necessary to further explore the relationship between ED consumption and quality of life, considering the increasing use of these products and their potential implications for physical, mental, and social health. While numerous studies have documented negative associations in adolescent populations, research focused on young adults and the general adult population remains scarce.

Given that the evidence has focused mainly on adolescents, it is necessary to study the relationship between energy drink consumption and quality of life in the Spanish adult population. In this context, this study examines the association between ED consumption and quality of life in Spanish adults, with a primary focus on sex differences; age is considered an essential descriptive and adjustment variable rather than a primary exposure. The research questions guiding this study are: Is there a significant relationship between ED consumption and perceived quality of life? Does this relationship vary according to sex? Addressing these questions will contribute to a deeper understanding of the phenomenon and generate evidence to inform health promotion and prevention strategies tailored to the characteristics of different population groups.

## Method

### Design

This is a descriptive, non-probabilistic study using a convenience sampling method. We conducted a cross-sectional study using an online questionnaire administered in Spain. The survey was hosted on Surveymonkey and took approximately 15 min to complete. All materials were in Spanish.

Recruitment followed a convenience, nationwide online strategy. We disseminated the survey link through university mailing lists and institutional channels, as well as social media (e.g., [X/Twitter, Instagram, WhatsApp, university websites]), enabling coverage across multiple regions of Spain. To minimize selection bias, invitations were worded broadly and did not mention hypotheses or expected outcomes.

Because data collection relied on online access, we restricted the target population to adults aged 18–54 years, an age band with consistently high internet penetration and digital literacy in Spain. Age ranges were established based on populations with adequate internet access, as indicated in the *Survey on Equipment and Use of Information and Communication Technologies in Households* (47). This choice minimizes mode-related noncoverage (limited internet access in older groups) while covering the younger adult segment in which ED use is more prevalent.

### Participants and eligibility criteria

Eligible participants were adults aged ≥18 years, residing in Spain, able to read Spanish, and who provided electronic informed consent. We excluded duplicate submissions (identified via timestamp/IP heuristics), respondents who failed attention checks, and records with missing key outcomes (WHOQOL-BREF domain scores). We did not exclude respondents on the basis of specific medical conditions; sensitivity analyses addressing self-rated health are reported in the Statistical Analysis section.

Before data collection, we planned to detect a small effect size for the association between energy drink (ED) consumption and WHOQOL-BREF domains while adjusting for 13 predictors (k = 13; ED [2 dummies], age [1], living arrangement [2], education [2], occupation [2], self-rated health [1], and past-month use of alcohol [1], tobacco [1], and cannabis [1]). As a pragmatic *a priori* rule for multiple regression, the minimum sample size to test the overall multiple correlation is N ≥ 50 + 8 m (48), where *m* is the number of predictors. With m = 13, this yields Ncalc = 50 + 8 × 13 = 154. (For testing individual predictors, the companion rule N ≥ 104 + m gives 117, also far below our final n.) Our achieved sample (n = 1,146) therefore provides ample power to detect small effects with adjustment for the specified covariates, ensuring precise estimates for the primary comparisons.

### Instruments

The first section of the survey included sociodemographic variables such as age, marital status, living arrangement, occupation, and perceived health status.

The substance use section included structured questions to assess the consumption of ED, alcoholic beverages, tobacco, and cannabis, based on items from the Spanish Survey on Drug Use in Secondary Education (ESTUDES) (49). The items estimating prevalence of use (alcoholic beverages, tobacco, cannabis, and energy drinks) were adapted from the standardized items employed by the Spanish Government Delegation for the National Plan on Drugs (DGPNSD) in its population surveys since 1995 (EDADES/ESTUDES). These items follow the harmonized methodology of the European Union Drugs Agency (EUDA; formerly EMCDDA) for consumption indicators (lifetime, past year, past month), with extensive use and temporal continuity in Spain. For this reason, because they are well-established and internationally comparable ítems, we did not conduct a separate, study-specific pilot for these questions; instead, we preserved the original wording and reference periods. For each substance, participants were asked about lifetime use, use in the past year, and use in the past month, as well as recent consumption frequency (“During the last 30 days, how often have you consumed [name of the substance]?”), with response options ranging from “None” to “Every or almost every day.”

Quality of life was assessed using the WHOQOL-BREF scale (50), widely applied in adult populations, developed and validated by the World Health Organization as the abbreviated version of the WHOQOL-100 questionnaire. This self-administered scale consists of 26 items: 2 general items regarding overall perception of quality of life and satisfaction with health (questions 1 and 2), and 24 items distributed across 4 domains: physical health, psychological health, social relationships, and environment. Each item is rated on a 5-point Likert scale reflecting intensity, capacity, frequency, or evaluation. Domain scores are linearly transformed to a 0–100 scale, where higher values indicate better quality of life.

### Statistical analysis

Statistical analyses were conducted using Stata v.17. A descriptive analysis of the sample and the prevalence of ED consumption was performed. Before conducting the analyses the database was cleaned through a series of of out-of-range and logical conditions, and a multiple imputation procedure was used to estimate missing values. To analyze the effect of ED consumption on scores from the different quality-of-life scales, we estimated linear regression models. Considering that the interaction between sex and ED consumption was significant, we opted to run sex-stratified estimates. We examined the distribution of residuals and homoscedasticity and, given evidence of some heteroskedasticity, used Huber–White robust variance estimators to preserve the validity of statistical inference. Moreover, because the sample size exceeds 50 participants, the normality assumption for residuals was not deemed critical, since by the central limit theorem the sampling distribution of the estimators approaches normality even when individual errors are non-normal. As a measure of model fit, we used the coefficient of determination (R^2^).

To assess the effect of daily ED consumption on respondents’ answers to specific quality-of-life items, we estimated cumulative odds via ordinal logistic regression models, also stratified by sex. To satisfy the proportional-odds assumption, the original five response options of the quality-of-life instrument were collapsed into three categories. Model fit was evaluated using the Cox–Snell (maximum likelihood) index.

In both the linear regression and ordinal logistic regression models, to control for potential confounding, we included the following as control variables: age, living arrangement, educational attainment, occupation, self-rated health, and past-month use of alcoholic beverages, tobacco, and cannabis.

### Ethical considerations

The study was approved by the Ethics Committee of the International University of Valencia (CEID2022_06 dated 27/05/2022).

## Results

Data were collected from 1,146 individuals (73.7% women, *n* = 845) aged between 18 and 54 years, with a mean age of 27.0 years (SD = 8.61). A total of 82.5% (*n* = 945) of the sample was under 34 years old (see Table 1).

**Table 1:** Sample description.

Variable	Sample (*n* = 1,146)
Age	
Years [mean (SD)]	27.0 (8.61)
Categorized age (years)	
< 25	650 (56.7)
25–34	295 (25.7)
35–44	124 (10.8)
45–54	77 (6.7)
Sex (female)	845 (73.7)
Highest level of education completed	
No formal education or primary education	11 (1.0)
Secondary education	586 (51.1)
University education	549 (47.9)
Living arrangement	
With family of origin	497 (43.4)
With own family	181 (15.8)
With partner	136 (11.9)
Shared accommodation	227 (19.8)
Lives alone	94 (8.2)
Other	11 (1.0)
Occupation	
Studies	471 (41.1)
Works	659 (57.5)
Other (unemployed, retired, or homemakers)	16 (1.4)
Perceived health	
Very good	177 (15.5)
Good	749 (65.4)
Fair	183 (16.0)
Poor or very poor	22 (1.9)
Energy drink consumption	
Ever used	718 (62.7)
Past year	441 (38.5)
Past month	244 (21.3)
Daily	65 (5.7)
Other substance use (past 30 days)	
Alcohol	880 (76.8)
Tobacco	431 (37.6)
Cannabis	183 (16.0)
Quality of life [mean (SD)]	
Quality of life (Item 1)	3.7 (0.87)
Satisfaction with health (Item 2)	3.5 (1.00)
Physical health (Domain 1)	68.2 (16.35)
Psychological health (Domain 2)	59.0 (18.66)
Social relationships (Domain 3)	64.6 (20.95)
Environment (Domain 4)	62.3 (15.60)

Variable distributions are presented as n (%), unless otherwise indicated.

Most participants reported having completed university (47.9%, *n* = 549) or secondary education (51.1%, *n* = 586). Considering the age distribution, the most common living arrangement was living with the family of origin (43.4%, *n* = 497) or sharing an apartment (19.8%, *n* = 227), followed by those living with their own nuclear family (15.8%). Regarding occupation, 57.5% (*n* = 659) report that they are working and 41.1% (*n* = 471) that they are studying.

Perceived health was mostly positive, with 81.9% (*n* = 926) rating it as “good” or “very good.” General quality of life received a mean score of 3.7, and satisfaction with health was rated at 3.5 (on a 5-point scale). Domain scores on the WHOQOL-BREF were also relatively high (on a 0–100 scale): physical health 68.2; psychological health 59.0; social relationships 64.6; and environment 62.3.

Regarding substance use, 62.7% (*n* = 718) of the sample reported having consumed ED at some point in their lives, 38.5% (*n* = 441) in the past year, 21.3% (*n* = 244) in the past month, and 5.7% (*n* = 65) reported daily consumption. In addition, 76.8% had consumed alcoholic beverages in the previous month, 37.6% (*n* = 431) reported tobacco use, and 16.0% (*n* = 183) cannabis use.

Table 2 presents the prevalence of ED consumption. A total of 21.3% (*n* = 244) reported occasional consumption, while 5.7% (*n* = 65) reported daily use. Prevalence was significantly higher among men (10.6% vs. 3.9% daily; and 30.6% vs. 18.0% occasional use). Additionally, 37.3% (*n* = 428) indicated they had never tried EDs (41.9% of women and 24.6% of men). Higher consumption rates were also observed among younger age groups. Among participants under 25 years of age, 6.5% (*n* = 42) reported daily consumption, compared to 5.4% (*n* = 16) in the 25–34 age group. This percentage decreased to 4.0% (*n* = 5) among those aged 35–44, and to 2.6% (*n* = 2) among individuals over 45 years of age.

**Table 2:** Prevalence of energy drink consumption.

Group	Never n (%)	Lifetime use (AVV) n (%)	Past 12 months (12 m) n (%)	Past 30 days (30d) n (%)	Daily use n (%)
Total	428 (37.3)	718 (62.7)	441 (38.5)	244 (21.3)	65 (5.7)
Women	354 (41.9)	491 (58.11)	279 (33.0)	152 (18.0)	33 (3.9)
Men	74 (24.6)	227 (75.4)	162 (53.8)	92 (30.6)	32 (10.6)
<25 years	230 (35.4)	420 (64.6)	301 (46.3)	175 (26.9)	42 (6.5)
25–34 years	89 (30.2)	206 (69.8)	107 (36.3)	52 (17.6)	16 (5.4)
35–44 years	59 (47.6)	65 (52.4)	24 (19.4)	12 (9.7)	5 (4.0)
45–54 years	50 (64.9)	27 (35.1)	9 (11.7)	5 (6.5)	2 (2.6)

AVV = ever in life; 12 m = past 12 months; 30d = past 30 days.

Among the total number of ED consumers (*n* = 718), 89.8% reported that on a typical day of consumption, they drink one serving (88.2% of women and 92.6% of men; see Table 3); 6.6% (*n* = 29) reported consuming two servings (6.8% of women and 6.2% of men); and 3.6% (*n* = 16) reported consuming three or more servings (5.0% of women and 1.2% of men).

**Table 3:** Number of energy drink servings on a typical day of consumption (among those reporting use in the past year).

Group	1 drink n (%)	2 drinks n (%)	≥3 drinks n (%)
Total	396 (89.8)	29 (6.6)	16 (3.6)
Women	246 (88.2)	19 (6.8)	14 (5.0)
Men	150 (92.6)	10 (6.2)	2 (1.2)
<25 years	272 (90.4)	19 (6.3)	10 (3.3)
25–34 years	96 (89.7)	6 (5.6)	5 (4.7)
35–44 years	22 (91.7)	2 (8.3)	0 (0.0)
45–54 years	6 (66.7)	2 (22.2)	1 (11.1)

The linear regression models estimating the relationship between ED consumption and quality of life, adjusted for potential confounders, indicate that the adjusted scores of ED consumers are similar in most cases to those of non-consumers (used as the reference group; Table 4), except for men in the mental health domain, whose score is significantly lower (−8.72 [95% CI − 16.999 to −0.45], *p* = 0.039). Although most associations did not reach statistical significance, the point estimates suggested lower scores in the physical and mental health domains, as well as in environment, compared with those who reported never consuming EDs, both among men and women and among occasional and daily ED consumers.

**Table 4:** Difference in scores across quality of life dimensions according to energy drink consumption (reference category: non-consumers of energy drinks).

Dimension	Sex	Lifetime use (AVV) score difference (95% CI)	Past 12 months (12 m) score difference (95% CI)	Past 30 days (30d) score difference (95% CI)	Daily use; score difference (95% CI)
Overall Quality of Life	W	−0.08 (−0.24 to 0.07)	−0.1 (−0.28 to 0.08)	−0.13 (−0.32 to 0.06)	−0.22 (−0.61 to 0.16)
	M	0.13 (−0.21 to 0.47)	−0.01 (−0.42 to 0.4)	−0.01 (−0.37 to 0.35)	−0.08 (−0.46 to 0.3)
Satisfaction with Health	W	−0.06 (−0.22 to 0.1)	−0,06 (−0,25 to 0,13)	0,05 (−0,15 to 0,24)	−0,31 (−0,75 to 0,14)
	M	0.09 (−0.33 to 0.51)	−0,07 (−0,49 to 0,35)	0,02 (−0,43 to 0,46)	−0,37 (−0,86 to 0,13)
Physical Health	W	−2,05 (−4,83 to 0,74)	−1.53 (−5.04 to 1.97)	−1.29 (−4.89 to 2.31)	−3.83 (−12.34 to 4.67)
	M	3,52 (−2,4 to 9,45)	2.61 (−3.48 to 8.71)	−2.68 (−9.66 to 4.31)	−3.45 (−10.35 to 3.45)
Mental Health	W	−1.9 (−5.14 to 1.34)	−1.5 (−5.47 to 2.48)	−2.04 (−6.41 to 2.32)	−4.73 (−13.03 to 3.58)
	M	0.33 (−6.25 to 6.9)	1.03 (−5.21 to 7.27)	−3.31 (−11.14 to 4.52)	**−8.72 (−16.99 to −0.45)** ***p 0.039 / R***^***2***^ ***0.1894***
Social Relationships	W	−1.22 (−5.15 to 2.72)	1.33 (−3.04 to 5.7)	0.47 (−4.57 to 5.5)	−3.28 (−13.82 to 7.26)
	M	4.88 (−3.21 to 12.96)	7.42 (−0.84 to 15.68)	3.23 (−5.36 to 11.83)	2.06 (−8.59 to 12.71)
Environment	W	−0.24 (−3.13 to 2.66)	0.5 (−3.09 to 4.08)	−0.34 (−3.82 to 3.14)	−7.68 (−15.65 to 0.29)
	M	−3.31 (−9.28 to 2.67)	−1.59 (−7.05 to 3.88)	−5.32 (−11.4 to 0.76)	−4.34 (−10.59 to 192)

The scale for items 1 and 2 (perceived quality of life and satisfaction with health) is 1–5, whereas the four domains (physical health, mental health, social relationships, and environment) are scaled 0–100. Linear regression models adjusted for age, living arrangement, educational attainment, occupation, self-rated health, and past-month use of alcoholic beverages, tobacco, and cannabis. H (men); M (women); ED (energy drinks); LT (lifetime); 12 m (past 12 months); 30d (past 30 days). *p*-value: two-sided significance of the t test of the estimated regression coefficient. R^2^: coefficient of determination. Bold formatting highlights statistically significant findings.

To further explore the perceived quality of life among the study population, selected items from the WHOQOL-BREF scale were analyzed (see Table 5). It can be seen that the cumulative odds (for daily consumers versus non-consumers) of responding affirmatively to the presence of negative feelings (3.53 [95% CI 1.28 to 9.75], *p* = 0.015) are significantly higher in men, but not in women. Men also show significantly higher odds of being dissatisfied with themselves (2.71 [95% CI 1.07 to 6.89], *p* = 0.036). Women, however, only exhibit significantly higher odds in perceiving a less healthy environment (2.58 [95% CI 1.28 to 5.17], *p* = 0.008).

**Table 5:** Cumulative odds of responses to selected quality of life scale items among daily energy drink consumers (reference category: non-consumers of energy drinks).

Item	Men odds (95% CI)	Women odds (95% CI)
Cumulative odds of responding affirmatively		
How often do you have negative feelings such as sadness, despair, anxiety, or depression?	**3.53 (1.28 to 9.75)** ***p 0.015/R***^***2***^ ***0.225***	1.09 (0.52 to 2.27)
Cumulative odds of responding negatively		
How satisfied are you with yourself?	**2.71 (1.07 to 6.89)** ***p 0.036/R***^***2***^ ***0.201***	1.66 (0.82 to 3.34)
How healthy is the environment around you?	1.97 (0.7 to 5.56)	**2.58 (1.28 to 5.17)** ***p 0.008/R***^***2***^ ***0.103***
How satisfied are you with your sleep?	1.18 (0.45 to 3.09)	0.98 (0.52 to 1.86)
How much do you enjoy life?	1.78 (0.49 to 6.45)	0.99 (0.31 to 3.13)
Do you have enough energy for daily life?	1.19 (0.48 to 2.99)	1.35 (0.64 to 2.81)
Are you able to accept your physical appearance?	0.86 (0.25 to 2.99)	1.72 (0.76 to 3.9)

Regression models adjusted for age, living arrangement, educational attainment, occupation, self-rated health, and past-month use of alcoholic beverages, tobacco, and cannabis. *p*-value: two-sided significance of the t test for the estimated regression coefficient. R^2^: model fit estimated using the Cox–Snell/ML index. Bold formatting highlights statistically significant findings.

## Discussion

The findings of this study confirm that ED consumption is particularly concentrated among males and younger age groups, which is consistent with previous studies identifying a higher prevalence among adolescents and young adults, along with a gender-based distribution (8–12). However, due to the cross-sectional nature of the study, the observed relationships between ED consumption and quality of life domains should be interpreted as associations, not causal effects.

Marketing also helps explain these differences. In Spain, as in other countries, advertising for EDs is primarily targeted at young adult men and links consumption with energy, masculinity, athletic performance, and fun (24, 25, 29, 30). This strategy fosters the normalization of use and reinforces the perception of “functional” benefits in academic, social, and sports contexts. From a public health perspective, these messages are a priority target for regulation due to their potential to entrench risk-prone attitudes and legitimize frequent ED consumption.

Although most individuals in this sample reported consuming a single unit per occasion, approximately 10% reported multiple units (6.6% two and 3.6% three or more), which could lead to caffeine intakes exceeding levels considered safe, especially among low–body-weight youth (4). Frequent ED consumption is consistently associated with sleep disturbances (shorter duration, poorer quality, insomnia, difficulty falling asleep, and other disorders; 16) (6–12, 26, 32). The likely mechanism includes the effects of caffeine and other stimulants, which disrupt circadian rhythms and impair sleep quality (51). Taken together, these alterations have negative repercussions for mental health and overall well-being (6, 7). The results of the present study are consistent with this evidence, as daily ED consumers reported dissatisfaction with sleep more frequently, especially men. These findings reinforce the need to consider the impact of ED consumption on sleep as a key mechanism in its relationship with perceived quality of life.

This exposure may also contribute to other adverse effects described in the literature. Several studies have documented associations between frequent ED consumption and physical discomfort, as well as psychological and physiological problems (9, 11, 34, 36–40), suggesting that beyond occasional use, habitual consumption may negatively affect perceived quality of life. Although our findings do not allow causal inferences, they align with prior evidence indicating a potentially harmful impact of ED consumption on well-being (9, 15, 16, 31–33).

In this study as well, daily ED consumers, compared to non-consumers, significantly higher odds of reporting negative feelings and dissatisfaction with themselves, particularly among males. Among females, a higher likelihood of perceiving their environment as less healthy was observed.

The analysis of specific WHOQOL-BREF items also showed that men who consume EDs daily are more likely to report negative moods and lower self-satisfaction compared to those who do not consume them. This trend is consistent with the literature, which has documented adverse effects of ED consumption on psychological health (6, 11, 27, 44).

Gender differences in perceived health and in ED consumption stem from a combination of social, cultural, physiological, and hormonal factors. Among women, taking on greater caregiving responsibilities and prolonged exposure to chronic stress have been associated with poorer perceived health and lower subjective well-being (52–54). This context may encourage the use of EDs as a strategy to cope with fatigue and stress; however, their physiological effects (e.g., sleep disturbances, nervousness) can paradoxically worsen the perception of an unhealthy environment and reduce quality of life (55). Moreover, sociocultural factors may heighten women’s awareness and critical appraisal of issues such as safety, social support, and domestic workload, leading to lower self-reported scores in health and environment domains when multiple stressors converge (56, 57).

In contrast, men more frequently show negative associations between ED consumption and mental health, likely mediated by masculine norms that promote vigor, risk-taking, and identification with models of physical or athletic toughness, behaviors often accompanied by higher caffeine and ED intake. This pattern has been linked in longitudinal studies to a progressive worsening of anxiety and depression symptoms (44, 58, 59).

These social dynamics are compounded by biological differences. Sex-based variations have been documented in responses to caffeine (pharmacokinetics, cardiovascular effects) and in subjective and physiological responses (60, 61), which could modulate the risk of adverse effects on mood and sleep; the evidence, however, is complex and not uniform. From a hormonal perspective, in women, fluctuations related to the menstrual cycle, pregnancy, or menopause may increase sensitivity to stress and fatigue, reinforcing both negative perceptions of the environment and the use of caffeine as a compensatory resource (60). In men, testosterone has been associated with greater sensation-seeking and risk-taking, which could help explain higher and more problematic ED use and, consequently, a less favorable mental health impact (17, 18). In any case, these biological differences interact closely with social and gender factors and should not be interpreted in isolation.

Finally, adult-focused motivational evidence complements these mechanisms. Studies in adults and young adults indicate motives centered on stress-coping and performance (e.g., studying, managing workload/fatigue), as well as social reasons (nightlife and alcohol-mixed use) (10, 27, 28, 55, 62). Social-context motives, including AmED (alcohol mixed with energy drinks), are documented and linked to risk behaviors (30, 63, 64). Taken together, these findings suggest a gendered motivational pattern: men may be more strongly driven by performance/energy and peer/social contexts (including AmED), whereas women may more often report stress- and fatigue-coping motives. This pattern aligns with the sex-specific associations observed in our study and provides a plausible behavioral pathway that complements our adjusted models.

The broader gender-and-health literature based on the WHOQOL-BREF shows consistent differences in perceived quality of life. In a study of 1,147 adults, Islam (65) found poorer outcomes among women in nearly all domains (physical, psychological, and environmental), except the social domain. Similar findings have been reported in other contexts: women showed poorer physical health and lower overall health satisfaction (66); female medical students scored lower than males in the physical and psychological domains (67); and female caregivers of people with rare diseases had lower scores in social relationships (68). Taken together, this evidence situates ED consumption within a broader framework of gender health inequalities and underscores the need to integrate biological and psychosocial determinants when analyzing its differential impact.

Finally, although the adjusted models did not show statistically significant differences across all quality of life domains, there is an average reduction of approximately 4–9 points in mental health and environment scores among daily consumers compared with non-consumers, in both men and women. These findings underscore the need for further longitudinal research on the effects of continued ED consumption in young adult and adult populations, and highlight the relevance of public health interventions aimed at regulating the marketing of these products and raising awareness of their potential risks.

### Preventive and public policy implications

Building on international experience, several policy levers have credible evidence and clear precedents. Nordic countries illustrate age-based access controls (e.g., sales bans to minors in Sweden and pharmacy-only dispensing in Norway), while Germany and Denmark have implemented statutory limits on caffeine/taurine content. In the United Kingdom, large supermarket chains have adopted voluntary under-16 sales restrictions reinforced by visible point-of-sale warnings and enhanced labeling. Drawing on these models (3, 69), our recommendations prioritize: (1) evaluating national age-of-sale restrictions; (2) mandating front-of-pack high-caffeine warnings and standardized per-serving caffeine caps; (3) restricting product placement and marketing in youth-dense environments (schools, sports venues, digital platforms); and (4) monitoring Alcohol-mixed-with-Energy-Drinks (AmED) practices and enforcing clear AmED warnings. Such measures are consistent with European policy trends and would reduce youth exposure and risk while supporting informed consumer choice.

Our findings strengthen the case for preventive and regulatory action, particularly among males and younger individuals, where prevalence is highest. The observed associations between frequent ED use and poorer perceptions of mental health, environment, and subjective well-being suggest that these drinks should not be considered harmless products. As highlighted by Harris and Munsell (29) and Visram et al. (12), aggressive youth-targeted marketing links EDs to culturally valorized attributes (performance, masculinity, fun, autonomy), underscoring the urgency of regulating advertising, labeling, and sales, especially in settings frequented by minors (schools, sports facilities, social media).

Given the widespread availability of EDs in supermarkets and vending machines without age restrictions, the legal framework warrants review. Potential measures include restricting sales to minors, requiring prominent warning labels, and limiting caffeine content per unit. In parallel, awareness campaigns aimed at young adults and families are recommended, as adult consumption can model and legitimize youth use. These campaigns should address the risks of regular ED consumption, the high caffeine and sugar content, and the hazards of combining EDs with alcohol. Incorporating ED-related content into school-based health education can further support informed decision-making about stimulant products and promote a culture of well-being and self-care.

Finally, these actions should be complemented by intersectoral strategies (health, education, community) to reduce adolescents’ exposure to and accessibility of EDs and to promote healthy, sustainable alternatives for enhancing personal well-being.

### Limitations

This study has several limitations that should be considered when interpreting the results. First, recruitment relied on convenience, online channels (university mailing lists and social media), which likely over-represent younger, more educated, and digitally active individuals; this selection pattern may bias prevalence estimates and limits external validity. Second, ED use and covariates were self-reported, making them susceptible to recall and social desirability biases; although the questionnaire was anonymous and neutrally worded to mitigate these issues, measurement error cannot be excluded. Third, the sample was not probabilistic and no post-stratification weighting was applied, so generalizability to the broader Spanish adult population is limited; future studies should replicate these analyses with larger, representative samples. Fourth, the cross-sectional design precludes causal inference (including potential reverse causation), and unmeasured confounding may partially explain some associations despite multivariable adjustment. Missing data were handled via complete-case analysis; missingness was low and reported in the Supplement, but residual bias remains possible.

Moreover, although data were collected on the consumption of other substances (alcohol, tobacco, and cannabis), these were not analyzed in relation to ED consumption or quality of life. This limits the understanding of potential patterns of polysubstance use and their cumulative or synergistic effects on adult and youth well-being. Given that previous research has identified links between ED consumption and other risk behaviors, such as the use of psychoactive substances (6), future studies should address these phenomena in an integrated manner.

Policy transferability may be constrained by differences in national regulatory frameworks and retail practices; thus, international examples should be contextualized to Spain’s legal and market environment.

In the present study, no statistically significant differences were observed in reports of physical pain interfering with the ability to perform certain activities, or in the acceptance of physical appearance, although a trend was noted in both men and women. While these findings should be interpreted with caution, they may reflect dimensions of physical and emotional discomfort that deserve further exploration in future studies with greater statistical power.

Finally, it is recommended that future research include longitudinal designs that allow for the analysis of ED consumption patterns over time, as well as their effects on physical, psychological, and social development. It would also be relevant to explore, from a developmental and gender-based perspective, the motives for consumption, risk perceptions, and the influence of the social and digital environment, in order to design more specific and effective interventions.

## Conclusion

This study provides empirical evidence on ED consumption and its relationship with perceived quality of life in young adult and adult populations. The findings reveal that 62.7% of the sample have consumed EDs at least once, 21.3% did so in the past month, and 5.7% consume them daily, especially among males and younger age groups, with a predominant pattern of occasional use, although not without cases of daily consumption and multiple servings per occasion.

Among young adult individuals (18–30 years), frequent ED consumption is significantly associated with poorer perceptions of mental health and environmental quality, particularly among those who report daily intake. This association is more pronounced in males, who exhibit higher consumption rates and a greater likelihood of experiencing emotional distress, personal dissatisfaction, and sleep-related difficulties. Among young adult females, frequent ED consumption is more clearly linked to negative evaluations of their environment, which may reflect a greater impact of social, academic, or relational factors.

In the adult population, although prevalence is lower, daily ED consumption is still associated with lower scores in mental health and environmental perception, suggesting that the adverse effects of these beverages are not limited to adolescence or early adulthood. The persistence of this pattern in older age groups underscores the need to consider ED consumption as a relevant public health issue beyond the school or youth context.

Overall, these results indicate that habitual ED use may negatively impact perceived well-being, particularly in terms of psychological health and social environment, and highlight the importance of implementing preventive strategies that address both access to these products and misconceptions about their effects. The observed differences by sex and age group emphasize the need for differentiated and context-sensitive interventions that take into account both individual and social factors influencing ED consumption.

Based on these findings, a coordinated strategy that takes into account age and sex is recommended: (1) education and health-promotion for young adults and families (school/university modules on stimulant use, sleep hygiene, and risks of alcohol-mixed energy drinks); (2) risk-proportionate marketing/sales regulation (youth-audience and placement restrictions, prominent front-of-pack high-caffeine warnings, standardized caffeine caps per serving); (3) consider age-of-sale limits and point-of-sale warnings; (4) clinical and public-health actions (routine screening in primary care and student health, brief advice for frequent users, targeted counseling for sleep/mental-health problems); and (5) monitoring and enforcement focused on AmED communications and sales. Together, these measures would support informed choices, curb risky patterns, and align Spain with emerging European best practices.

## Data Availability

The raw data supporting the conclusions of this article will be made available by the authors, without undue reservation.
